# Circular RNA hsa_circ_0001306 Functions as a Competing Endogenous RNA to Regulate FBXW7 Expression by Sponging miR-527 in Hepatocellular Carcinoma

**DOI:** 10.7150/jca.61381

**Published:** 2021-09-09

**Authors:** Yufan Wu, Taihe Fan, Yubin Zhao, Rongkuan Hu, Dongdong Yan, Ding Sun, Ling Gao, Lei Qin, Xiaofeng Xue

**Affiliations:** 1 Department of General Surgery, The First Affiliated Hospital of Soochow University, Suzhou 215000, JiangSu Province, China.; 2 Department of Biochemistry, College of Life Sciences, Shaanxi Normal University, Xi'an, China.; 3 College of Life Sciences, University of Science and Technology of China, Hefei, China.; 4 Kunshan Hospital of Traditional Chinese Medicine, Kunshan, JiangSu Province, China.; 5 Department of General Surgery, Changshu NO.1 People's Hospital Affiliated to Soochow University, Changshu, JiangSu Province, China.

**Keywords:** Hepatocellular carcinoma, Hsa_circ_0001306, Cell proliferation and invasion, miR-527, F-box and WD repeat domain containing 7

## Abstract

Hepatocellular carcinoma (HCC) is one of the most common types of cancer worldwide. Circular RNAs (circRNAs) have been reported to regulate many types of cancers, including HCC. The purpose of this study was to investigate the potential roles of hsa_circ_0001306 in HCC. Firstly, the downregulation of hsa_circ_0001306 was identified by high‑throughput RNA sequencing and further verified by qRT-PCR. Secondly, we evaluated the effects of hsa_circ_0001306 on HCC cell proliferation, invasion, cell cycle. Finally, we used an animal model to validate the *in vitro* experimental results. The expression of hsa_circ_0001306 was closely related to tumor size. Knockdown of hsa_circ_0001306 could downregulate F-box and WD repeat domain containing 7(FBXW7), a target of miR-527, thereby promoting HCC cell proliferation and invasion. Furthermore, hsa_circ_0001306 siRNA increased the multiplication rate of HCC tumors. Mechanistic studies indicated that hsa_circ_0001306 acts as a ceRNA for miR-527, which resulted in the reduction of its endogenous target, FBXW7. Hsa_circ_001306 is significantly downregulated in HCC, and the hsa_circ_0001306/miR-527/FBXW7 axis plays an important role in HCC progression.

## Introduction

Hepatocellular carcinoma (HCC) is one of the most common types of cancer worldwide. More than 750,000 individuals are diagnosed with this disease annually 1. Although progress has been made in its diagnosis and treatment, which includes surgical techniques and liver transplantation, the long-term survival rate of patients with HCC remains very low. Therefore, new biomarkers and therapeutic targets are needed to improve the outcome of patients with HCC 2.

circRNA is a type of non-coding RNA, whose study has recently gained momentum 3. Compared with traditional linear RNA, circRNA has a closed circular structure, which is free from the influence of RNA exonucleases. Therefore, circRNA expression is stable and not easily degraded 4-7. Recent studies have shown that circRNAs, which are rich in microRNA (miRNA) binding sites, function as miRNA sponges to remove the inhibitory influence of miRNAs on their target genes 8. Therefore, by means of the competitive endogenous RNA (ceRNA) mechanism, circRNAs function as important regulators in various diseases 9.

MicroRNAs (miRNAs) are highly conserved endogenous RNAs 20-24 nucleotides in length, which play a significant role in regulating the target mRNAs. The deregulation of miRNAs is usually related to the tumor development in multiple human cancers including HCC. MiR-527 was involved in several cancers 10, 11, such as osteosarcoma and non-small-cell lung cancer. For instance, miR-527 could inhibit TGF-β/SMAD signaling pathway via decreasing the expression of SMAD4 and TβRII (TGFBR2).

FBXW7 (F-box and WD repeat domain containing 7) is a member of the F-box protein family, which is an essential tumor suppressor and is frequently inactivated in human cancer cells including HCC 12. FBXW7 expression is reduced in hepatocellular carcinoma (HCC) tissues. However, the tumorigenic roles and mechanisms for miR-527 and FBXW7 in HCC development and progression remain unknown.

In the present study, we identified a novel HCC-related circRNA, hsa_circ_0001306, which was significantly downregulated in HCC specimens. The expression of hsa_circ_0001306 was closely related to tumor size. Moreover, we found that hsa_circ_0001306 may function as a ceRNA for miR-527, thereby reducing the level of its endogenous target, F-box and WD repeat domain containing 7 (FBXW7). Therefore, hsa_circ_0001306 may serve as a new target for HCC.

## Materials and methods

### Patients and clinical specimens

Fifty HCC specimens and paired adjacent tissues were collected from patients who underwent hepatectomy at the First Affiliated Hospital of Soochow University from January 2014 to December 2016. All patients did not receive chemotherapy or radiotherapy before surgery. Based on the evaluations of experienced pathologists, the paired adjacent non-tumor tissues were harvested from the tumor edge at 5 cm, and no visible tumor cells were found. All tissue specimens were stored in liquid nitrogen. Clinical information was obtained under the study protocol approved by the Research Ethics Committee of the First Affiliated Hospital of Soochow University and written informed consent was obtained from each subject.

### High‑throughput RNA sequencing

The mirVana miRNA Isolation Kit (Ambion, Austin, Texas, USA) was used to extract the total RNA. The Agilent 2100 Bioanalyzer (Agilent Technologies, Santa Clara, CA, USA) was used to assess RNA integrity. The libraries sequenced on the Illumina sequencing platform (HiSeqTM 2500 or another platform) were instituted by TruSeq Stranded Total RNA with Ribo-Zero Gold.

### Cell culture

Four hepatoma cell lines (SK-HEP-1, Hep-3B, Hep-G2, and HUH-7), the normal hepatic cell line THLE-2, and the 293T cell line were purchased (6-12 months prior to experiments) from Type Culture Collection of the Chinese Academy of Science (Shanghai, China). All cell lines were analyzed by the short tandem repeat STR method recommended by American Type Culture Collection (ATCC) when we purchased. All six cell lines were cultured in Dulbecco's Modified Eagle Medium (DMEM) supplemented with 10% fetal bovine serum (FBS) in humidified air containing 5% CO_2_ at 37 °C 13-14.

### RNA extraction and qRT-PCR assays

We extracted total RNA from each HCC specimen and paired paracancerous liver tissue using Ezol Reagent (Genepharma, Shanghai, China) according to the manufacturer's instructions. The purity and quality of total RNA was assessed using the NanoDrop 2000 Spectrophotometer (Thermo Fisher Scientific, Wilmington, DE, USA). We synthesized the target cDNA by reverse transcription (RT) using random primers and the GoScript RT System (Promega, Madison, WI, USA). A non-template reaction was served as the control. The GoTaq qPCR Master Mix (Promega) and the qRT-PCR Plus System (Stratagene, La Jolla, CA, USA) were used for the real-time quantitative reverse transcription-polymerase chain reaction (qRT-PCR). Primers for hsa_circ_0001306 and glyceraldehyde 3-phosphate dehydrogenase (G3PDH) were synthesized by GenePharma (Shanghai, China). To validate the hsa_circ_0001306 was circular, the extracted total RNA was incubated at 37℃ with or without RnaseR (3U/ug) for 30 minutes to digest the linear RNA. The hsa_circ_0001306 expression level was then detected with its specific primers. The sequences of G3PDH, hsa_circ_0001306, miR-527, U6, and FBXW7 primers were in Supplementary File 3.

### Cell transfection

To alter the expression of the target gene, cells were transfected with siRNAs and the negative control (NC) using Lipofectamine 2000 (Invitrogen, Carlsbad, CA, USA) according to the manufacturer's instructions. The hsa_circ_0001306 siRNA, which targets the junction region of the hsa_circ_0001306 sequence, was designed and synthesized by GenePharma. The miR-527 mimic, miR-527 inhibitor, and scrambled negative control siRNA (NC) were designed and synthesized by GenePharma. The sequences were as follows:

- hsa_circ_0001306 siRNA-1: sense 5'-GAUACAUUUCUAUUCCCCATT-3'; antisense 5'-UGGGGAAUAGAAAUGUAUCTT-3';
- hsa_circ_0001306 siRNA-2: sense 5'-CAUUUCUAUUCCCCAGGAATT-3'; antisense 5'-UUCCUGGGGAAUAGAAAUGTT-3';
- miR-527 mimic: 5'-CUGCAAAGGGAAGCCCUUUC-3'; and NC: 5'-AGUCGUCUAUAGAAGUUCGAGC -3';
- miR-527 inhibitor: 5'-GAAAGGGCUUCCCUUUGCAG-3'; and, NC: 5'-UCUGCACUAUUCAAGUGUGACC -3'.

### Cell counting kit-8 (CCK-8) assay

Transfected cells (5 × 10^3^ cells/well) were seeded in a 96-well plate and cultured for 0, 24, 48, and 72 h. Thereafter, Cell Counting Kit-8 Reagent (Dojindo, Japan) was added to each well, and the plate was incubated for approximately 2 h. The absorbance was measured in a microplate reader at 450 nm.

### Colony formation assay

Transfected cells were seeded in a 6-well plate and cultured in DMEM supplemented with 10% FBS in humidified air containing 5% CO_2_ at 37 ºC for ten days. Cells were fixed with methanol for 30 min and stained with crystal violet (Beyotime, Shanghai, China) for 30 min. This experiment was performed three independent times to reduce variations.

### Transwell invasion assay

To evaluate the invasion capacity of the transfected cells, Transwell chambers (Corning, Corning, NY, USA) were used. Transfected cells (1 × 10^5^ cells) were added into the upper chamber with a Matrigel-coated membrane (BD Biosciences, Franklin Lakes, NJ, USA). DMEM supplemented with 10% FBS was added into the lower chamber. After incubation for 36 h, cells that invaded the Matrigel and migrated to the underside of the Transwell chamber membrane were fixed and stained with crystal violet for 30 min. The number of stained cells was counted in five randomly selected visual fields under a Leica DM3000 microscope.

### Ethynyldeoxyuridine (EdU) analysis

We analyzed the proliferative capacity of HCC cells using the Cell-Light^TM^ EdU Apollo567 *In vitro* Kit (RiboBio, Guangzhou, China). Transfected cells were incubated with 5 μM EdU according to the manufacturer's instructions. Thereafter, the cells were fixed in 4% formaldehyde for 30 min and permeabilized with 0.5% Triton-X100 for 10 min. EdU-positive cells were examined under a fluorescence microscope in dark conditions.

### Flow cytometric analysis

Transfected cells were stained with fluorescein isothiocyanate-Annexin V and propidium iodide and analyzed by flow cytometry (BD Biosciences). Transfected cells were stained with the CycleTEST™ PLUS DNA Reagent Kit (BD Biosciences) according to the manufacturer's instructions. To examine the cell cycle, cells were analyzed by flow cytometry. The relative number of cells in G0/G1, S, and G2/M phases was determined and compared between the groups.

### Xenograft tumor model construction in nude mice

For this experiment, ten male BALB/c-A nude mice purchased from the Shanghai Laboratory Animal Experimental Animal Center of the Chinese Academy of Sciences were used. After subcutaneous incubation of HCC tumors for seven days, we injected 10 OD cholesterol-modified hsa_circ_0001306 siRNA-1 or control siRNA every three days for five weeks. The tumor size was measured (tumor volume, V = 0.5 × L × W2) twice a week. Finally, we removed and weighed the subcutaneous tumors. We performed all animal experiments according to the Animal Management Rules of the Chinese Ministry of Health (document 55, 2001).

### Protein extraction and Western blot assay

We extracted total proteins from HCC cells using RIPA extraction reagent (Beyotime, Shanghai, China) supplemented with a protease inhibitor cocktail (Roche). We collected the supernatants after centrifugation at 16000 g and 4 °C for 15 min. The total proteins were separated by 12% SDS and transferred onto polyvinylidene fluoride membranes (Millipore, Burlington, MA, USA). The membranes were blocked with 5% low fat powdered milk for 1 h and incubated with primary antibodies (Abcam, Cambridge, MA, USA) overnight at 4 °C. The immunoreactive proteins were detected by the electrochemiluminescence detection system (Thermo Fisher Scientific).

### Fluorescence *in situ* hybridization (FISH)

The expression levels and locations of hsa_circ_0001306 and hsa-miR-527 were detected by FISH in HCC cell lines and tumor tissues obtained from nude mice. The sequences of the probes were as follows:

- hsa_circ_0001306: GTGATGGC+TTCCTGGGGA+ATAGAAATGTATCC+T+AGGCT and,
- hsa-miR-527: GAAAG+GGCTTCCC+TTTGCAG.

### Luciferase assay

The hsa_circ_0001306 fragments containing the putative binding sites of miR-527 and its mutant sequence were synthesized and cloned into the luciferase reporter gene psiCHECK2 (Promega) and designated hsa_circ_0001306-WT and hsa_circ_0001306-Mut, respectively. The vectors were sequenced and respectively co-transfected with miR-527 or miR-NC into 293T cells. After co-transfection for approximately 48 h, luciferase activity was measured using the Dual Luciferase Reporter Assay Kit (Promega, Shanghai, China).

### RNA pull down

Firstly a biotinlabeled miR-527 probe was synthesized by GenePharma Co.,Ltd (Suzhou, China) to bind to the possible binding sites of hsa_circ_0001306. The probe sequence was Bio-5'-CTGCAAAGGGAAGCCCTTTC-3'. The antisense of miR-527 was used as negative control (NC). The NC sequence was Bio-5'-GAAAGGGCTTCCCTTTGCAG-3'.Approximately 1×10^7^ miR-527-overexpressing Hep1 cells were washed with ice-cold phosphate-buffered saline (PBS), lysed in RNA lysis buffer and centrifuged then. The supernatant was incubated with streptavidin magnetic Dynabeads (M-280, Invitrogen) to enrich the miR-527 probe overnight at 30 °C. Later the probes-dynabeads-miRNAs mixture was washed and the RNA was extracted using TRIzol Reagent and the content of hsa_circ_0001306 was analyzed by qRT-PCR.

### Statistical analysis

Student's *t*-test was used to analyze differences between the two groups. The Pearson correlation coefficient was used to indicate the relationship between hsa_circ_0001306 and miR-527. GraphPad Prism 6.0 Software (GraphPad Software Inc., La Jolla, CA, USA) was used to analyze and present the data. All data are expressed as mean ± standard deviation (SD). *P*-values < 0.05 were considered statistically significant.

## Results

### Identification of hsa_circ_0001306 as a downregulated circRNA in HCC

**Figure 1:**
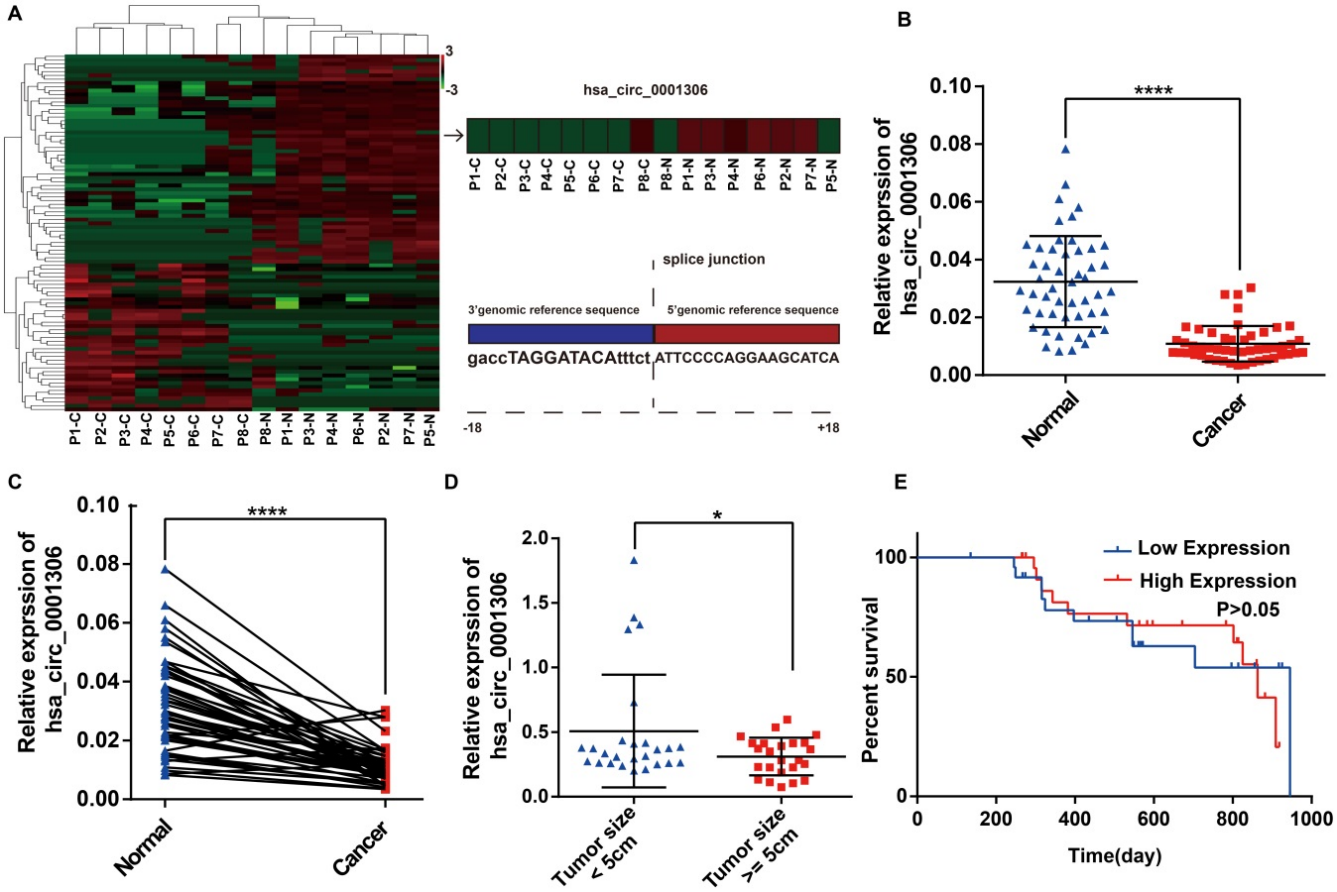
** Downregulation of hsa_circ_0001306 in HCC tissues and cell lines. A.** Heatmap shows 96 differently expressed circRNAs from high‑throughput RNA sequencing. The splice junction is listed in Fig 1A. **B and C.** The relative expression of hsa_circ_0001306 in HCC specimens and adjacent normal tissues from 50 patients is shown in B and C. The expression of hsa_circ_0001306 is significantly downregulated in HCC specimens (****, *P* < 0.0001). **D.** The expression of hsa_circ_0001306 is significantly decreased in patients with tumor size ≥ 5 cm (*, *P* < 0.05). **E.** Kaplan-Meier analysis shows no difference between hsa_circ_0001306 high and low expression groups.

To identify differentially expressed circRNAs between HCC specimens and paired adjacent normal tissues, eight pairs of specimens (eight HCC specimens and eight matched non-tumor liver tissues) were analyzed by high throughput RNA sequencing. As shown in the heatmap (Figure 1A), 40 upregulated circRNAs and 56 downregulated circRNAs were identified between HCC and adjacent normal specimens by high throughput RNA sequencing (fold-changes >2.0, P-values <0.05). We found that the hsa_circ_0001306 expression level was significantly downregulated in tumor tissues compared with adjacent normal tissues. The origin of has_circ_0001306 is RAD54L2. To validate these results, we conducted qRT-PCR to quantify hsa_circ_0001306 expression among 50 pairs of HCC and paired adjacent normal specimens. As shown in Figure 1B and C, hsa_circ_0001306 expression was significantly decreased in HCC specimens compared with normal liver tissues. The expression was downregulated more significantly when the tumor size was ≥ 5 cm compared to when it was < 5 cm (Figure 1D). However, the overall survival time between the high and low expression groups was not different (Figure 1E) (P > 0.05).

### Knockdown of hsa_circ_0001306 promotes HCC proliferation *in vitro* and *vivo*

**Figure 2:**
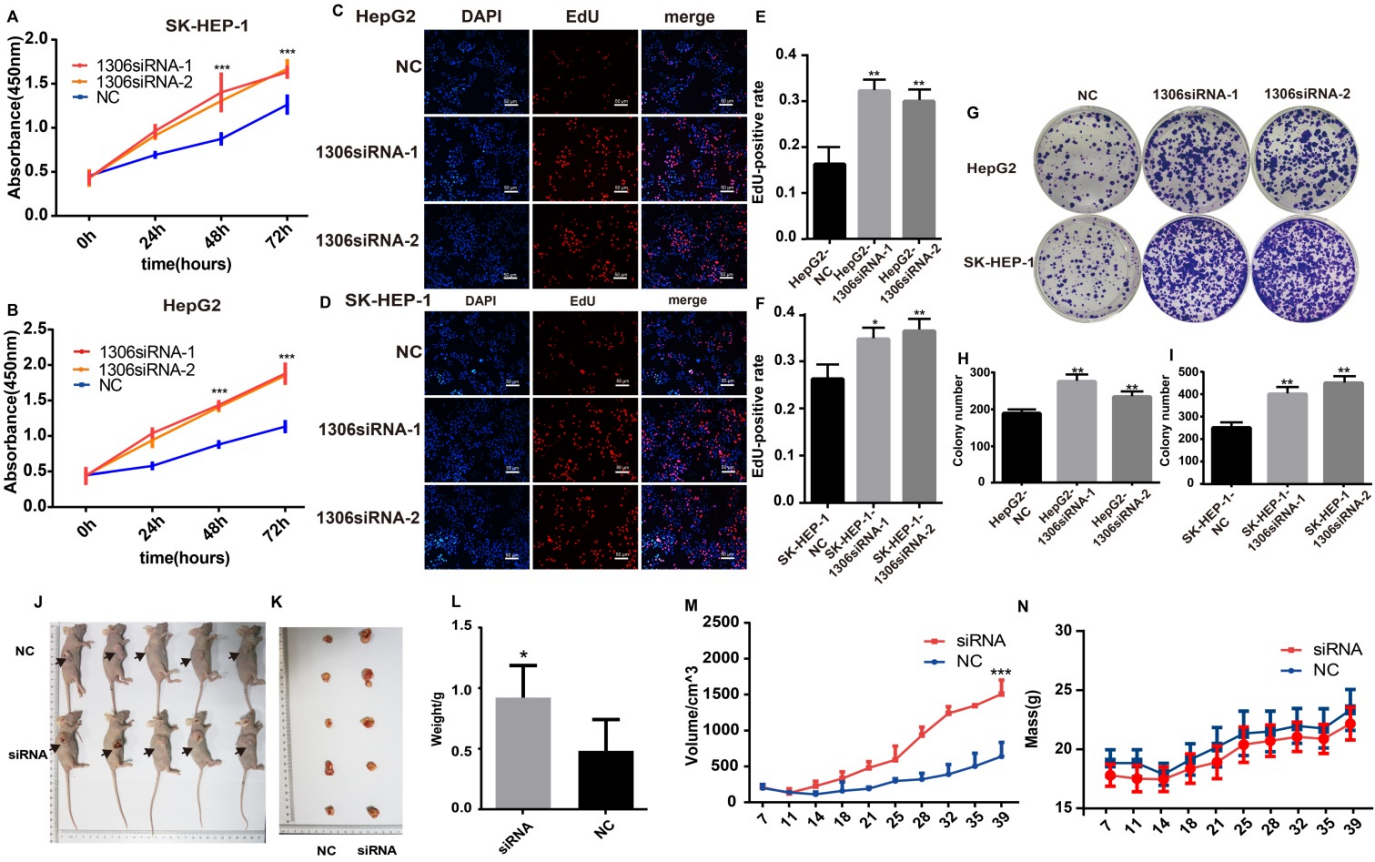
** Hsa_circ_0001306 knockdown promotes HCC proliferation *in vitro* and *vivo*. A and B.** CCK-8 assay shows the different proliferative capability of cells in the NC group and the hsa_circ_0001306 siRNA groups (***, *P* < 0.001). **C-F.** EdU analysis of proliferating cells in the siRNA groups (original magnification, 100×; scale bars, 50 µm; **, *P* < 0.01; *, *P* < 0.05) compared with the NC group. **G-I.** Colony formation assays demonstrating the proliferative capability of cells in the siRNA groups (**, *P* < 0.01), compared with the NC group. **J-L** Comparison of nude mouse tumor specimens between experimental and NC groups on day 39 after injection. **M.** Tumor volumes were measured twice a week (***, *P* < 0.001). **N.** The body weights were measured twice a week.

Hep-G2 and SK-HEP-1 cells were selected for further experiments because they had the highest expression of hsa_circ_0001306, as confirmed by qRT-PCR (Supplementary File 1). The expression of hsa_circ_0001306 was significantly decreased in both hsa_circ_0001306 siRNA-1 and hsa_circ_0001306 siRNA-2 groups (Supplementary File 2A-B). Several experiments were then carried out to validate the roles of hsa_circ_0001306 in cell proliferation and invasion. Short-term cell proliferative capability was determined with the CCK-8 assay. As shown in Figure 2A and B, Hep-G2 and SK-HEP-1 cell proliferation was higher in both hsa_circ_0001306 siRNA-1 and hsa_circ_0001306 siRNA-2 groups than that in the NC group. To validate the effect of hsa_circ_0001306 on cell proliferation, the EdU assay was performed. The results showed that DNA synthesis was significantly enhanced in the hsa_circ_0001306 siRNA-1 and hsa_circ_0001306 siRNA-2 groups. EdU positive rate was higher in both the hsa_circ_0001306 siRNA-1 and hsa_circ_0001306 siRNA-2 groups compared with that in the NC group (Figure 2C-F). Moreover, both Hep-G2 and SK-HEP-1 cell lines transfected with either hsa_circ_0001306 siRNA-1 or hsa_circ_0001306 siRNA-2 formed significantly more and bigger colonies than NC-transfected cells in colony formation assays, which is consistent with the aforementioned findings. The results of the colony formation assay illustrated that hsa_circ_0001306 siRNA-1 and hsa_circ_0001306 siRNA-2 could promote long-term HCC cell proliferation (Figure 2G-I). To investigate whether hsa_circ_0001306 affects HCC growth *in vivo*, we inoculated Hep-G2 cells subcutaneously into male nude mice. All mice developed tumors. After continuous intra-tumoral injection of cholesterol-conjugated hsa_circ_0001306 siRNA-1 for six weeks, we found that tumor growth was significantly enhanced in the experimental group. The average size and weight of tumors in the hsa_circ_0001306 siRNA-1 group were significantly larger than those in the control group (Figure 2J-N).

### Knockdown of hsa_circ_0001306 promotes the invasion, reduces the ratios of SK-HEP-1 and Hep-G2 cells in G0/G1

**Figure 3:**
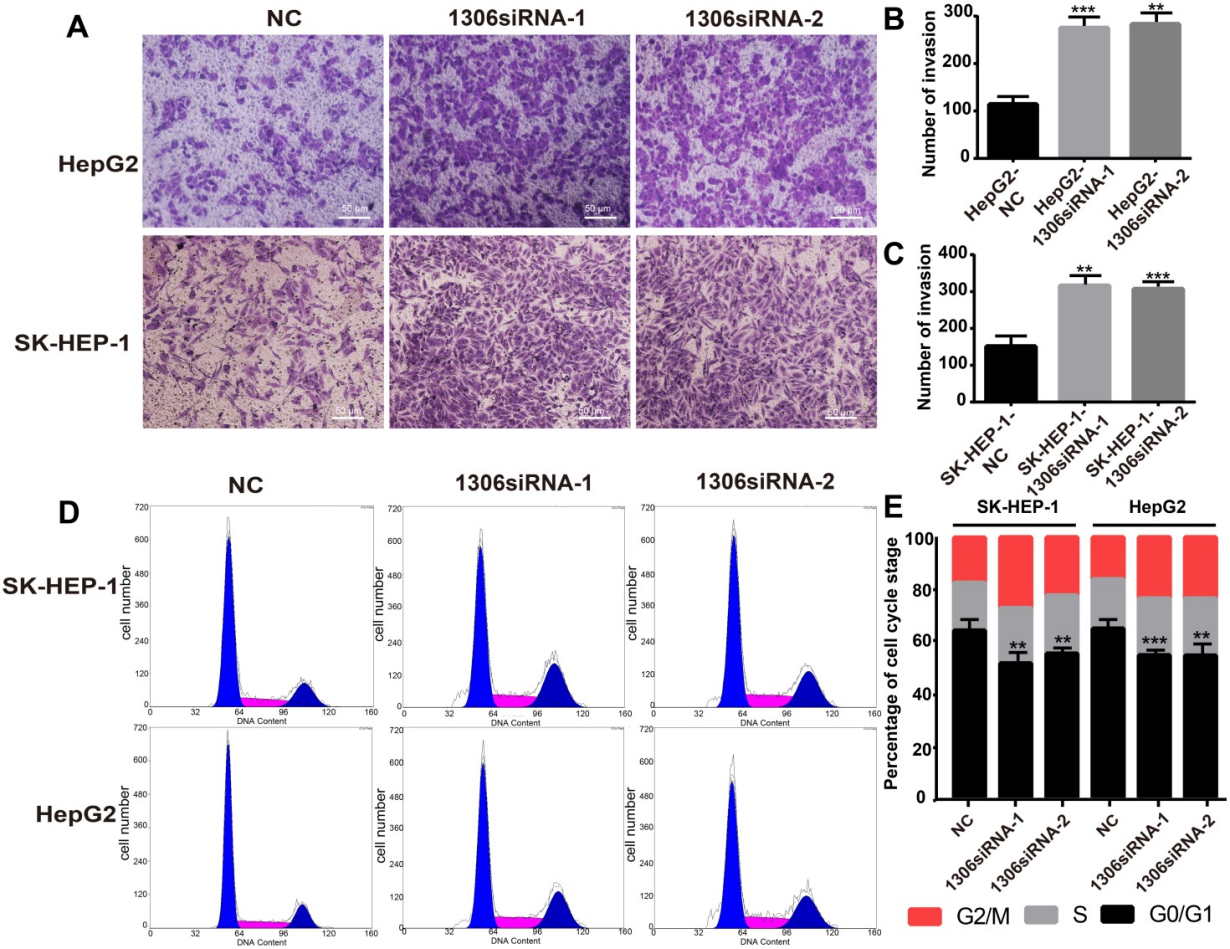
** Hsa_circ_0001306 siRNA promotes the invasion, reduces the ratios of SK-HEP-1 and Hep-G2 cells in G0/G1 *in vitro*. A-C.** Invasive capability of cells in the siRNA groups compared with the NC group in Transwell invasion assays (×100; scale bars, 50 µm). The number of invasive cells was shown for each group (***, *P* < 0.001; **, *P* < 0.01). **D and E.** There were fewer transfected SK-HEP-1 and Hep-G2 cells in the G0/G1 phase of the cell cycle compared with control cells (***, *P* < 0.001; **, *P* < 0.01).

The invasion ability and cell cycle are key indicators of tumor cell proliferation. Therefore, to investigate whether hsa_circ_0001306 could affect HCC cell invasion, we performed an invasion assay with transfected HCC cells. The results showed that Hep-G2 and SK-HEP-1 cell invasion was enhanced in hsa_circ_0001306 siRNA-1 and hsa_circ_0001306 siRNA-2 groups compared with the NC group (Figure 3A-C). Besides, the ratios of Hep-G2 and SK-HEP-1 cells transfected with si-circRNA-1306 in G0/G1 significantly decreased. Compared with control cells, fewer cells were in the G0/G1 phase, whereas more cells were in the S/G2 phase (Figure 3D, E).

### Hsa_circ_0001306 functions as a ceRNA to sponge miR-527 in HCC cells

**Figure 4:**
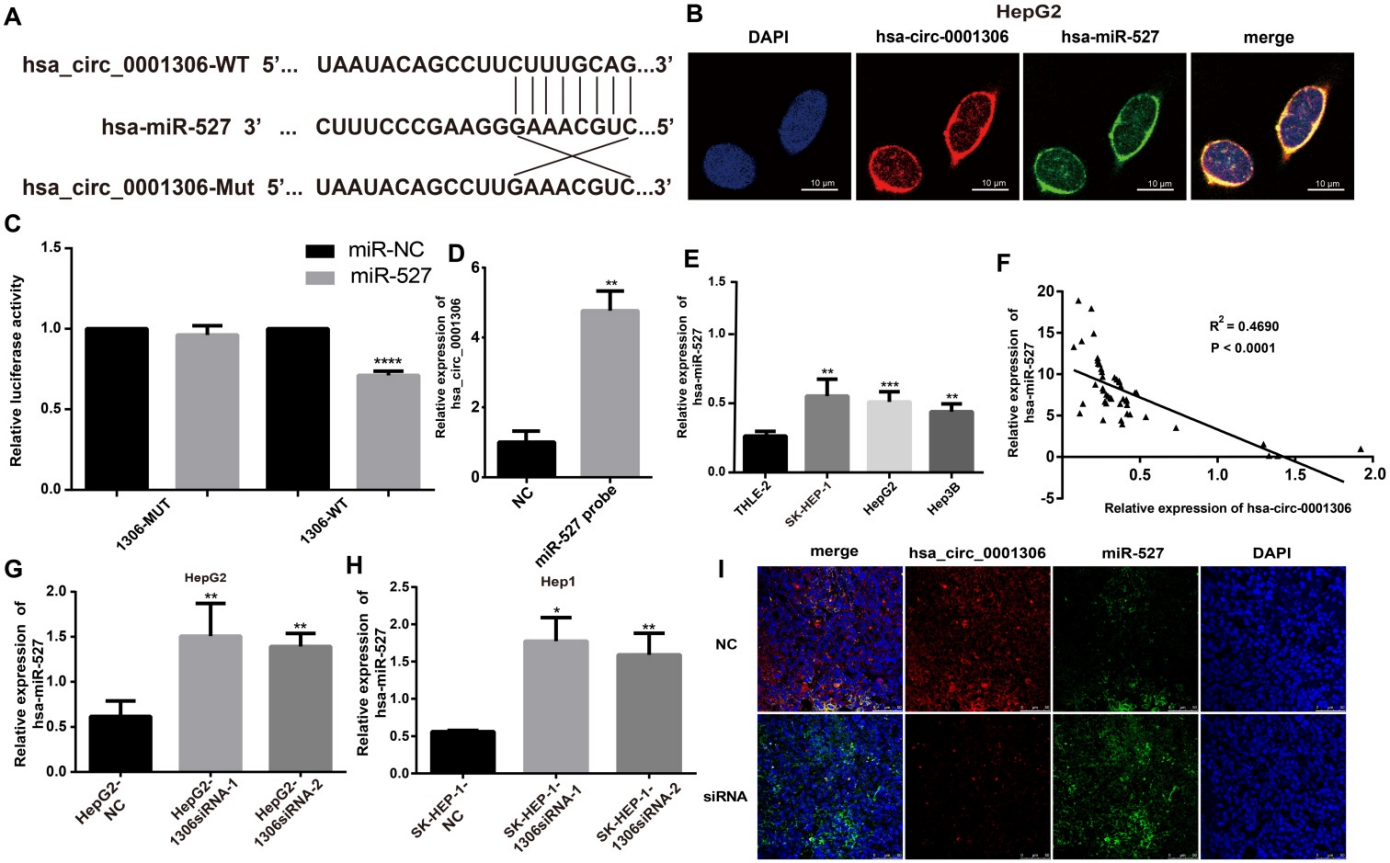
** MiR-527 is a direct target of hsa_circ_0001306. A.** Predicted miR-527 seed region at the wild-type (WT) and mutated hsa_circ_0001306 3'UTR. **B.** FISH analysis showing the location of hsa_circ_0001306 and miR-527 in HepG2, which is the most abundant in the cytoplasm (scale bars, 10 µm). **C.** Luciferase reporter assay of 293T cells co-transfected with the miR-527 mimic and either hsa_circ_0001306/3'-UTR-WT or hsa_circ_0001306/3'-UTR-Mut. **D.** The RNA pull down assay. The miR-527 probe group is different from the NC group (**, *P* < 0.01). **E.** MiR-527 expression levels in different cell lines (****, *P* < 0.0001). **F.** A negative correlation between miR-527 and hsa_circ_0001306 is shown by Spearman's rank correlation analysis in HCC specimens. **G and H.** qRT-PCR analysis of miR-527 expression levels in Hep-G2 and SK-HEP-1 cells transfected with hsa_circ_0001306 siRNA-1 or hsa_circ_0001306 siRNA-2 (**, *P* < 0.01; *, *P* < 0.05). **I.** FISH assay indicating the location and expression of hsa_circ_0001306 and miR-527 in siRNA and NC tissues obtained from the nude mouse tumor specimens (original magnification, 100×; scale bars, 50 µm).

Bioinformatics tools analysis revealed the potential binding site on hsa_circ_0001306. The top five miRNAs scored by bioinformatics tools are miR-15a-3p, miR-527, miR-1972, miR-500a-3p and miR-593-5p. Based on the results of bioinformatics prediction analyses 15-17, we hypothesized that one of these miRNAs might be a target gene of hsa_circ_0001306. Therefore, a luciferase expression assay was performed to test this postulate. Finally, mir-527 was found to be the most possible target of hsa_circ_0001306. In brief, 293T cells were co-transfected with the miR-527 mimic and the wild-type or mutated plasmid or the negative control. The wild-type binding sites within the hsa_circ_0001306' untranslated region (UTR) of 293T cells co-transfected with the miR-527 mimic resulted in a significant difference in the luciferase activity compared with control cells, whereas there was no difference after co-transfection with the miR-527 mimic and the mutated plasmid compared with that of the control cells (Figure 4A, C). Moreover, the RNA pull down shows similar result (Figure 4D). The miR-527 probe group is different from the NC group.

We analyzed the locations of hsa_circ_0001306 and miR-527 by the FISH assay. The results revealed that hsa_circ_0001306 and miR-527 were the most abundant in the cytoplasm (Figure 4B), suggesting that hsa_circ_0001306 and miR-527 might affect each other in this cellular compartment. To further analyze the relationship between miR-527 and hsa_circ_0001306, we examined miR-527 expression level in 50 paired tissues, human hepatocytes, and four HCC cell lines by qRT-PCR. We found that miR-527 expression in human hepatocytes was lower than that in the three HCC cell lines (Figure 4E). Furthermore, miR-527 and hsa_circ_0001306 expressions were negatively correlated in the paired tissues (Figure 4F). To further validate the relationship, qRT-PCR was used to analyze hsa_circ_0001306 expression in Hep-G2 and SK-HEP-1 cells transfected with hsa_circ_0001306 siRNA-1 or hsa_circ_0001306 siRNA-2. Indeed, miR-527 expression was higher in hsa_circ_0001306 siRNA-1 and hsa_circ_0001306 siRNA-2 groups than that in the NC group (Figure 4G, H). Moreover, the results of FISH analysis in nude mouse tumor specimens showed that hsa_circ_0001306 expression was significantly decreased in the experimental group compared with that of the control group, whereas miR-527 expression was significantly increased in the experimental group (Figure 4I). In addition, hsa_circ_0001306 co-localized with miR-527 in the cytoplasm. Taken together, these results indicate that hsa_circ_0001306 can negatively regulate hsa-miR-527 expression.

### Knockdown of miR-527 inhibits the invasion and increases the ratios of SK-HEP-1 and Hep-G2 cells in G0/G1

**Figure 5:**
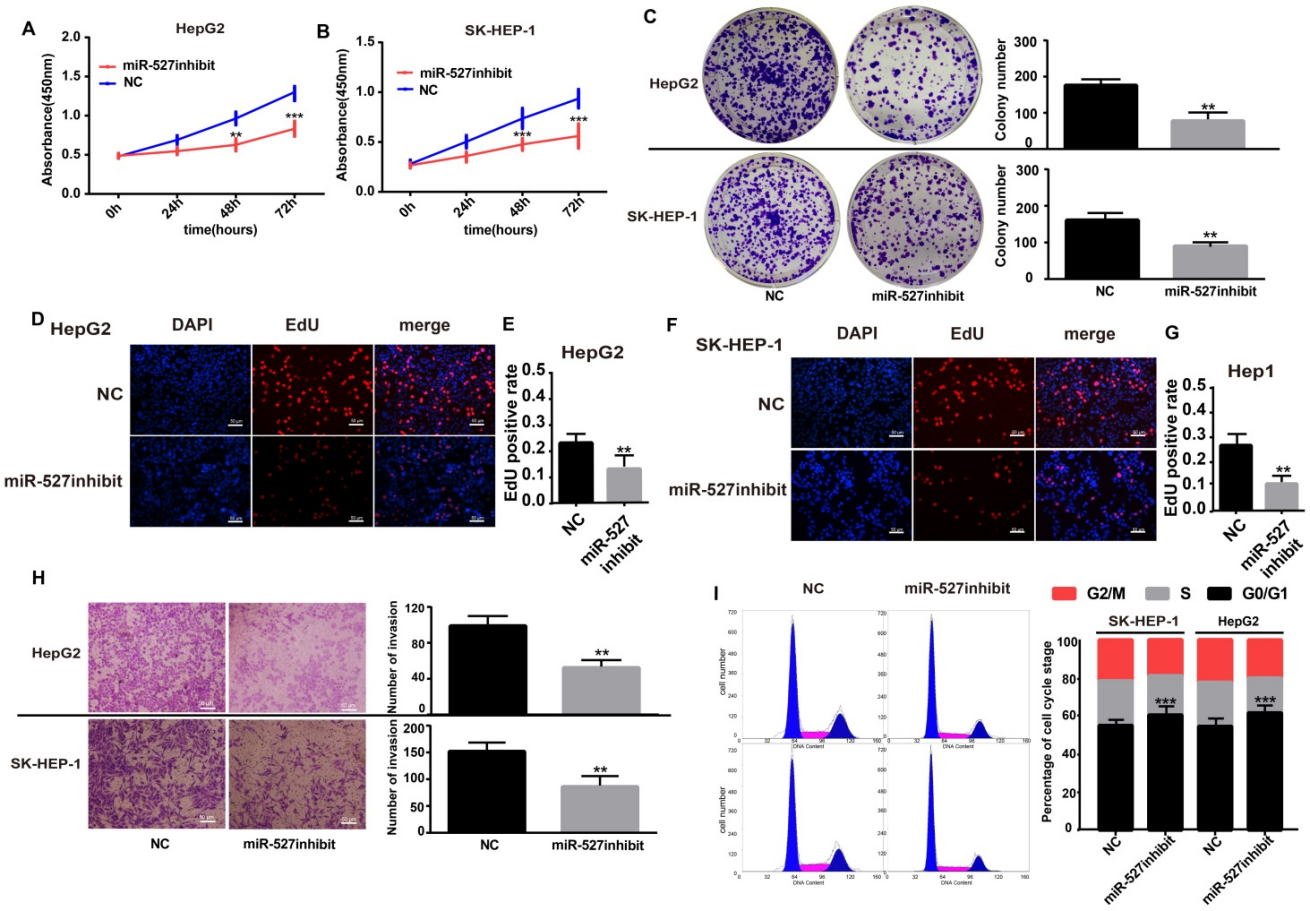
** Influence of miR-527 inhibit on HCC cell proliferation, invasion and cell cycle *in vitro*. A and B.** Growth curves for Hep-G1 and SK-HEP-1 cells after transfection with the miR-527 inhibit or control miRNA by the CCK8 assay (***, *P* < 0.001; **, *P* < 0.01). **C.** Colony formation assays demonstrating the proliferative capability of cells in the miR-527 inhibit group (**, *P* < 0.01) compared with control cells. **D-G.** EdU analysis of proliferating cells in the miR-527 inhibit group (original magnification, 100×; scale bars, 50 µm; **, *P* < 0.01) compared with control Hep-G2 and SK-HEP-1 cell lines. **H.** Transwell assay showing the invasive capacity of the miR-527 inhibit group (×100; scale bars, 50 µm). The number of invasive cells per field is shown (**, *P* < 0.01; ***, *P* < 0.001). I. There are more miR-527 inhibit transfected SK-HEP-1 and Hep-G2 cells in the G0/G1 phase of the cell cycle than NC cells (***, *P* < 0.001).

Although miR-527 is confirmed to be negatively regulated by hsa_circ_0001306, but it is still not known whether miR-527 is a downstream effector of hsa_circ_0001306. Toward this end, we performed several assays, including cell proliferation, cell invasion and cell cycle in Hep-G2 and SK-HEP-1 cells by transfecting with the miR-527 inhibit. The expression of hsa-miR-527 was significantly affected in both miR-527 inhibit and miR-527 mimic groups (Supplementary File 2C-F). As shown in Figure 5A and B, cell proliferation was inhibited in the miR-527 inhibit group compared with the NC group via CCK-8 assays (Figure 5A, B). In addition, cell proliferation was further validated by colony formation and EdU assay. To our predicted miR-527 inhibit could significantly suppress Hep-G2 and SK-HEP-1 cell colony formation and EdU incorporation (Figure 5C-G). Furthermore, transwell assay and flow cytometric assays showed opposite results of knockdown of hsa_circ_0001306 siRNAs. After transfection of miR-527 inhibit, cell invasion was weakened and the ratios was increased in both Hep-G2 and SK-HEP-1 cells (Figure 5H-I).

### FBXW7 is a miR-527 target gene that can be suppressed by hsa_circ_0001306 siRNAs

**Figure 6:**
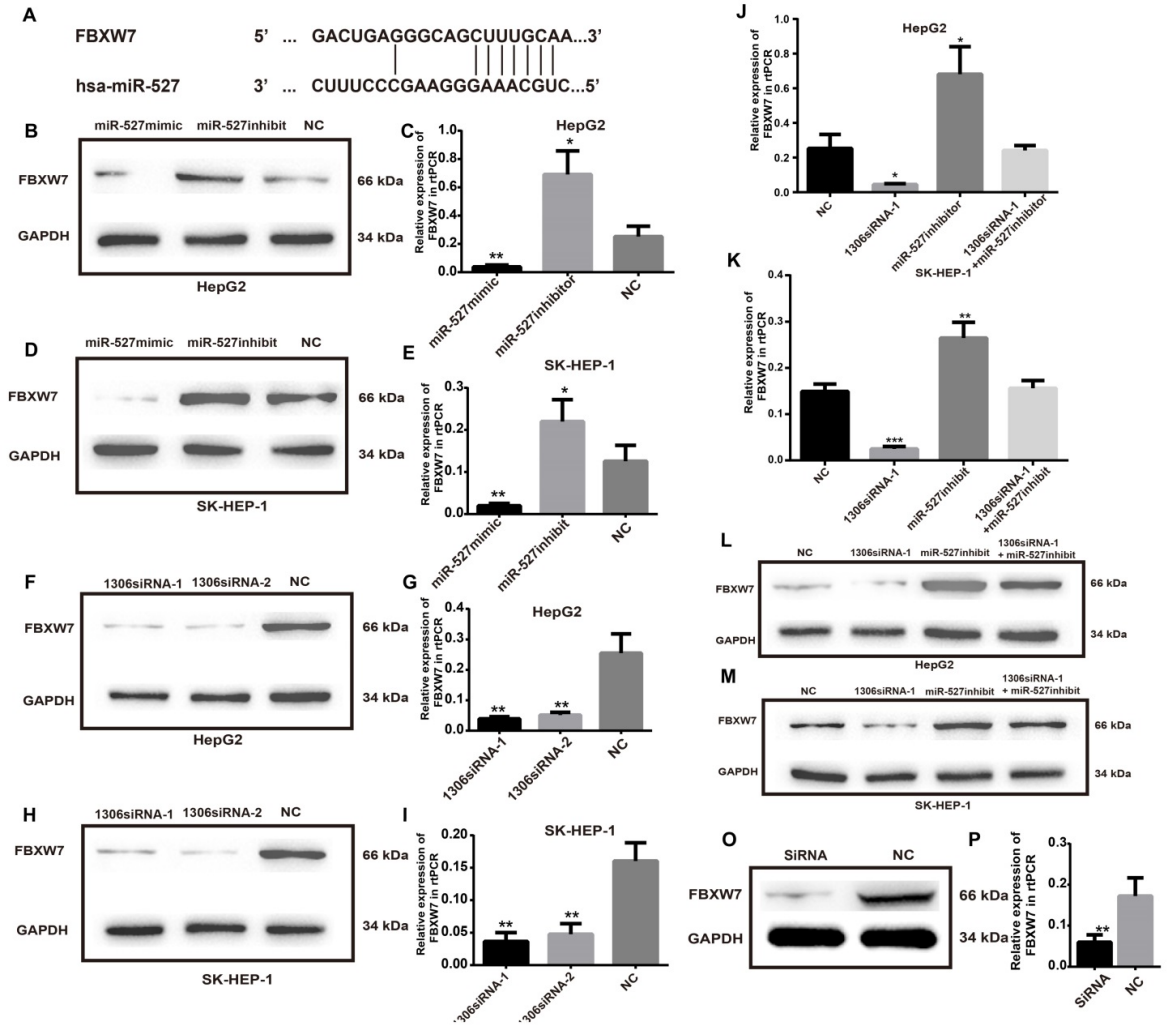
** FBXW7 is a target of miR-527 and is suppressed by hsa_circ_0001306-siRNA1 and siRNA 2. A.** Predicted miR-527 seed region in FBXW7 3'UTR. **B-E.** FBXW7 mRNA and protein level in Hep-G2 and SK-HEP-1 cells transfected with the miR-527-mimic, miR-527-inhibitor, and control miRNA. **F-I.** Relative FBXW7 mRNA and protein levels in Hep-G2 and SK-HEP-1 cells transfected with the hsa_circ_0001306 siRNA-1 and hsa_circ_0001306 siRNA-2. **J-M.** FBXW7 mRNA and protein levels in Hep-G2 and SK-HEP-1 cells after the knockdown of hsa_circ_0001306 and/or miR-527 inhibitor (***, *P* < 0.001; **, *P* < 0.01; *, *P* < 0.05). O-P. Relative FBXW7 mRNA and protein levels in nude mouse tumor specimens (experimental and NC groups; **, *P* < 0.01).

To investigate the detailed mechanism of miR-527 in HCC tumorigenesis, we first measured the expression of FBXW7, a direct target of miR-527 predicted by the miRBD 18, 19 (Figure 6A). As shown in Figure 6B-E, the transfection of miR-527 mimic significantly decreased the mRNA and protein expression levels of FBXW7 in Hep-G2 and SK-HEP-1 cells. On the contrary, the miR-527 inhibitor could increase the mRNA and protein expression levels of FBXW7 in Hep-G2 and SK-HEP-1 cells. Furthermore, to clarify whether hsa_circ_0001306 play roles in HCC via sponge of miR-527 and by means of FBXW7, we then detected the mRNA and protein expression levels of FBXW7 in Hep-G2 and SK-HEP-1 cells after transfection of siRNAs targeting hsa_circ_0001306 (Figure 6F-I). Then, we also examined HCC cells co-transfected with hsa_circ_0001306 siRNA-1 and the miR-527 inhibitor. Not surprisingly, the suppression of FBXW7 mRNA and protein levels by hsa_circ_0001306 siRNA-1 was effectively reversed by the miR-527 inhibitor (Figure 6J-M). Moreover the mRNA and protein levels of FBWX7 were downregulated in nude mouse tumor specimens (Figure 6O-P). Collectively, hsa_circ_0001306 suppresses HCC tumorigenesis by regulating miR-527 and FBXW7 pathway.

## Discussion

Although great strides have been made on the effective and accurate diagnosis and treatment of HCC, the high malignant level of HCC is a huge challenge in clinical practice. Therefore, novel biomarkers and therapeutic targets are urgently required to improve the outcome of HCC patients.

Increasing evidences have recently indicated that circRNAs are critically important in cell activities and cancer development. Progressive and uncontrolled tumor growth can be attributed to the dysregulation of circRNAs 20, 21. In the present study, we identified hsa_circ_0001306, which was significantly downregulated in HCC specimens and cell lines. A low expression level of hsa_circ_0001306 was associated with a larger tumor size of HCC. In addition, *in vitro* and *in vivo* experiments have confirmed that knockdown of hsa_circ_0001306 promoted cell proliferation and invasion. Consistent with previous studies, our findings indicated that hsa_circ_0001306 served as a ceRNA to exert its biological function in the development of HCC through a circRNA/miRNA/mRNA axis 22, 23.

**Figure 7:**
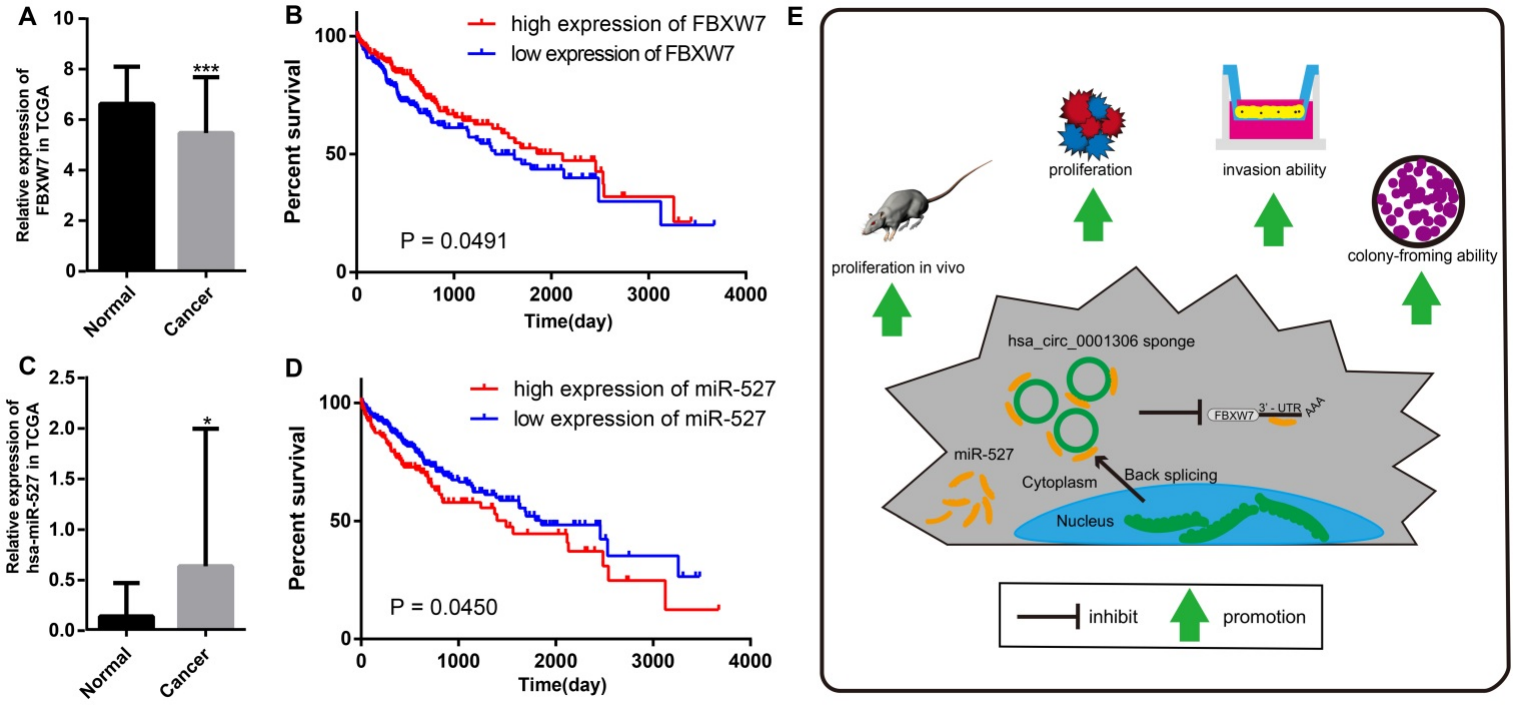
** The relative expression and clinical data of miR-527 and FBXW7 in TCGA and the schematic plot for hsa_circ_0001306/miR-527/FBXW7 pathway.** The relative expression of FBXW7 and miR-527 in HCC specimens and adjacent normal tissues from TCGA is shown in **A and C**. The expression of miR-527 is significantly upregulated in HCC specimens (*, *P* < 0.05). The expression of FBXW7 is significantly downregulated in HCC specimens (***, *P* < 0.001). **B.** Kaplan-Meier analysis shows significant difference between FBXW7 high and low expression groups (P = 0.0450). **D.** Kaplan-Meier analysis shows significant difference between miR-527 high and low expression groups (P = 0.0491). E. Schematic representation of the interplay among RNAs in the hsa_circ_0001306/miR-527/FBXW7 pathway.

We further proved that miR-527 was the miRNA target of hsa_circ_0001306 by bioinformatic prediction and dual-luciferase reporter assay, which was mainly distributed in the cytoplasm. miR-527 was significantly upregulated in HCC specimens, and it promoted cell proliferation by downregulating FBXW7. Similarly, miR-527 was also upregulated in HCC samples obtained from TCGA compared with that of normal tissues (Figure 7A). Moreover, Kaplan-Meier survival analysis on HCC samples from TCGA showed that miR-527 was unfavorable to the overall survival of HCC patients, manifesting as a better prognosis in HCC patients expressing a low level of miR-527 (Figure 7B). Several miRNAs have been identified upregulated in multiple types of cancers, which are served as oncogenes 24. Yang et al. found that miRNA-92a promotes the growth of HCC by targeting FBXW7. In addition, miRNA-149* can promote cell proliferation and inhibit apoptosis of T-cell acute lymphoblastic leukemia. Through downregulating ABCG2, hsa-miR-520h inhibits cell migration, invasion, and side populations from pancreatic cancer 26-28. Therefore, the novel regulatory mechanism of circRNA/miRNA/mRNA axis is believed to remarkably influence the development of HCC.

Bioinformatic analysis showed that FBXW7 was a novel target of miR-527, which was significantly downregulated in HCC tissues compared with that of normal tissues in TCGA. Kaplan-Meier survival analysis on HCC samples from TCGA concluded that FBXW7 was favorable to the prognosis of HCC (Figure 7C and D). Overexpression of miR-527 downregulated both mRNA and protein levels of FBXW7 in HCC cells. As a member of the F-box protein family, FBXW7 is an important tumor-suppressor gene and one of the most-commonly deregulated ubiquitin-proteasome system proteins in human cancers 12. The degradation of oncoproteins, such as cyclin E, c-Myc, Mcl-1, mTOR, Jun, Notch, and AURKA, can be regulated by FBXW7 29,30. As a frequently-mutated gene in cancers, we identified that FBXW7 was downregulated in HCC specimens compared with that of normal tissues. c-Myc and cyclin E are substrates of FBXW7, which are negatively correlated with the protein expression of FBXW7 in HCC tissues 31. Moreover, a low protein expression of FBXW7 is associated with poor pathological features, including a large tumor size, high pathological grading, and advanced TNM staging 32. A previous study has shown that FBXW7 predicts favorable 5-year overall survival and disease-free survival of HCC patients through analyzing TCGA dataset, suggesting that FBXW7 can be an independent prognostic factor for HCC 33. Our studies indicated that hsa_circ_0001306 exerted the miRNA sponge effect on miR-527 as a ceRNA, thus reducing the inhibitory effect of miR-527 on its target gene FBXW7, and as a result, FBXW7 was upregulated in HCC.

There are several limitations in our research. Firstly, although hsa_circ_0001306 was downregulated in HCC cells, its full-length was too long and it was difficult being artificially overexpressed. Therefore, we selected HCC cell lines expressing a relatively high level of hsa_circ_0001306 to carry out the knockdown experiments. hsa_circ_0001306 overexpression models established by novel technologies need to be further investigated. Secondly, we confirmed that miR-527 was the downstream target of hsa_circ_0001306 involved in the regulation of HCC development through mediating proliferation and invasion. Other existing miRNAs involved in should also be explored in the future. The development of HCC is mediated through complicated genetic regulatory systems involving interactions between DNAs, RNAs, proteins, and small molecules. Although our findings revealed the critical function of hsa_circ_0001306 in regulating proliferation and invasion of HCC, Kaplan-Meier survival curves did not identify the prognostic potential of it in HCC patients. A further larger study among HCC patients will be needed in future research. Thirdly, a total of 96 differentially expressed circRNAs between HCC and adjacent normal specimens from TCGA were identified by high throughput RNA sequencing (fold-change >2.0, *P*-value <0.05), and expression levels of the top 10 circRNAs were examined in 50 pairs of HCC and normal tissues collected in our center by qRT-PCR. We finally selected hsa_circ_0001306 that possessed the most significant *P* value for the following experiments. The potential functions of other significantly differentially expressed circRNAs in HCC need further research.

## Conclusions

In summary, we are the first to identify a novel HCC-related circRNA, hsa_circ_0001306, and to reveal that hsa_circ_0001306 is a tumor suppressor gene that can inhibit cell proliferation and invasion by the miR-527-FBXW7 axis in HCC. A schematic representation of the interplay among RNAs within the hsa_circ_0001306/miR-527/FBXW7 pathway is shown in Figure 7E. Our findings strengthen our understanding of the ceRNA mechanism of circRNAs in HCC progression, and hsa_circ_0001306 may be a potential biomarker for HCC diagnosis and treatment.

## Supplementary Material

Supplementary figures and table.

## Abbreviations

HCC: Hepatocellular carcinoma
CircRNAs: Circular RNAs
FBXW7: box and WD repeat domain containing 7
ceRNA: competitive endogenous RNA
MiRNAs: MicroRNAs
ATCC: American Type Culture Collection
DEME: Dulbecco's Modified Eagle Medium
FBS: fetal bovine serum
qRT-PCR: real-time quantitative reverse transcription-polymerase chain reaction
G3PDH: glyceraldehyde 3-phosphate dehydrogenase
NC: negative control
EdU analysis: Ethynyldeoxyuridine analysis
FISH: Fluorescence *in situ* hybridization
CCK-8 assay: Cell counting kit-8 assay
